# Supplementary material for: A general orientation distribution function for clay-rich media
Source: Nat Commun. 2019 Nov 29;10:5456. doi: 10.1038/s41467-019-13401-0 (PMC6884531; doi:10.1038/s41467-019-13401-0)
Supplement: Supplementary file 2 — Supplementary Information [file 41467_2019_13401_MOESM2_ESM.pdf]

# Supplementary Information for

# A general orientation distribution function for clay-rich media

Dabat et al.

## Table of Contents

**Supplementary Note 1**. Relation between the angle τ on the detector and the orientation angle $\theta$ of the normal to a clay platelet and implications on the extracted values of order parameters ……………….2

**Supplementary Fig. 1**. Geometric description of the X-ray scattering experiment ……………….......3

**Supplementary Fig. 2**. Assessment of the $\theta$=$\tau$ approximation ……………………………………......4

**Supplementary Fig. 3**. Influence of the $\theta$=$\tau$ approximation on the calculated order parameter values…………………………………………………………………………………………........4

**Supplementary Note 2**. Correlation between Lagrange multipliers $\lambda_2$ and $\lambda_4$ …………………….....5

**Supplementary Fig. 4**. Correlation between $\lambda_2$ and $\lambda_4$ Lagrange multipliers ………………………..5

**Supplementary Note 3**. Experimental assessment of sample slicing along the main stratigraphic direction….……………………………………………………………………………………...……5

**Supplementary Fig. 5**. Experimental assessment of sample slicing along the main stratigraphic direction……………………………………………………………………………………..……..6

**Supplementary Note 1. Relation between the angle τ on the detector and the orientation angle θ of the normal to a clay platelet and implications on the extracted values of order parameters.**

The geometrical description of the X-ray experiments we performed is summarized in Supplementary Fig. 1 within the framework of the Ewald sphere construction. Notations are detailed in Supplementary Fig. 1. In brief, the Ewald sphere construction is a geometric construction which allows one to visualize the occurrence of Bragg peaks. One draws a sphere of radius $2\pi/\lambda$ (the incident wave-vector modulus) around the sample. The origin of the reciprocal space is at the intersection of the transmitted X-ray beam and of this sphere. The conservation of wave-vectors implies that Bragg peaks are observable whenever a reciprocal lattice point lies exactly on the Ewald sphere.

The orientation of the normal $\vec{n}$ to a platelet with respect to the mean orientation axis *z* is given by an angle *θ*, as shown in Fig. 1a. The *001* Bragg reflections from all the platelets, whose orientations are given by the orientation distribution function *f(θ)*, result in a ring on the planar detector. The position of a point *P* on this ring is given by the angle τ between *Cz* and $\overrightarrow{CP}$, and the intensity at point *P* is written $I(\tau)$. There is a one-to-one correspondence between point *P* and a wave-vector $\vec{Q}_{001} \equiv \overrightarrow{OM}$ on the front of the Ewald sphere. This wave-vector is parallel to the normal $\vec{n}$ of diffracting platelets; thus, one finds the following simple relation:

$$I(\tau) \propto f(\theta) \quad (1)$$

In the (O*xyz*) frame and using Supplementary Fig. 1, the relationship between the angles $\theta$ and $\tau$ is obtained as follows:

$$\vec{Q}_{001} \equiv \overrightarrow{OM} = \frac{2\pi}{\lambda}(\cos(2\theta_B), \sin(2\theta_B)\sin(\tau), \sin(2\theta_B)\cos(\tau)) \quad (2)$$

where $\theta_B$ is the Bragg angle verifying the well-known relation:

$$Q_{001} = \frac{4\pi}{\lambda}\sin(\theta_B) \quad (3)$$

Projection of $\vec{Q}_{001}$ on the Oz axis thus also leads to:

$$Q_{001,z} = \frac{4\pi}{\lambda}\sin(\theta_B)\cos(\theta) \quad (4)$$

Following Supplementary Eq. (2) and Supplementary Eq. (4), $\frac{2\pi}{\lambda}\sin(2\theta_B)\cos(\tau) = \frac{4\pi}{\lambda}\sin(\theta_B)\cos(\theta)$, wherefrom the general relation between the angles $\theta$ and $\tau$ is as follows:

$$\cos(\theta) = \cos(\tau)\cos(\theta_B) \quad (5)$$

For sufficiently small values of $\theta_B$, its cosine can be approximated as 1 so that Supplementary Eq. (5) gives:

$$\theta = \tau \quad (6)$$

Taking into account the normalization of the ODF in Eq. (3), one thus obtains Eq. (4):

$$f(\theta) = \frac{I(\theta)}{\int_0^\pi I(\theta)\sin(\theta)d\theta}. \quad (7)$$

To validate the $\theta$=$\tau$ approximation in Supplementary Eq. (6), let us consider the $Q_{001}$ values at 0.4, 0.42, 0.44, 0.63, and 0.88 Å$^{-1}$ for smectite, vermiculite, chlorite, mica, and kaolinite, respectively. The worst scenario corresponds to the highest $Q_{001}$ value (i.e., $Q_{001}$=0.88 Å$^{-1}$ for kaolinite). When $\lambda$=1.5418 Å, $\theta_B \approx 6.2°$ and Supplementary Eq. (5) becomes:

$$\cos(\theta) = 0.994\cos(\tau) \quad (8)$$

Supplementary Fig. 2 reports $\theta = \mathrm{f}(\tau)$ and shows that $\theta$ can only be considered equal to $\tau$ in the range from ~10 to ~170°. Moreover, the diffraction condition, which is the location of the wave-vector $\vec{Q}_{001}$ on the Ewald sphere in Supplementary Fig. 1, cannot be satisfied when $\theta < \theta_B$, which is also evidenced in Supplementary Fig. 2. Consequently, even if one takes into account the exact relation between $\theta$ and $\tau$ in Supplementary Eq. (5), our X-ray scattering experiments will only give access to the ODF in a limited range between $\theta_B$ and 180°-$\theta_B$, i.e., between 6.3 and 173.7° in the present case.

To evaluate the effect of the approximation we used in the article by taking $\theta$=$\tau$ between 0 and 180°, let us consider the following general form for the intensity distribution:

$$I(\tau) \propto \exp(\lambda_2 P_2(\cos(\tau)) + 0.005(\lambda_2)^5\, P_4(\cos(\tau))) \quad (9)$$

which is based on the general ODF we determined for clay platelets in Eq. (21) using the maximum-entropy method. Let us now consider the exact relation between $\theta$ and $\tau$ for $\theta \in [\theta_B, \pi - \theta_B]$, so that according to Supplementary Eq. (1) and Supplementary Eq. (5), the ODF is written as:

$$f(\theta) \propto \exp(\lambda_2 P_2(\cos(\theta)\,/\cos(\theta_B)) + 0.005\,\lambda_2^5 P_4(\cos(\theta)\,/\cos(\theta_B))) \quad (10)$$

Let us also assume that $f(\theta) = f(\theta_B)$ for $\theta < \theta_B$ or $\theta > \pi - \theta_B$, which will lead to an underestimation of the calculated order parameters. The calculated order parameters $\langle P_2 \rangle$, $\langle P_4 \rangle$, and $\langle P_6 \rangle$ are compared for the worst scenario (i.e., for kaolinite) in Supplementary Fig. 3 to those calculated using the ODF in Eq. (21) with $\theta$=$\tau$ between 0 and $\pi$. The very good agreement observed fully justified our approximation. A reason for such a good agreement is the presence of the sinus of the $\theta$ angle in the calculation of the order parameters (Eq. (10)), which minimizes the contributions close to 0 and $\pi$.

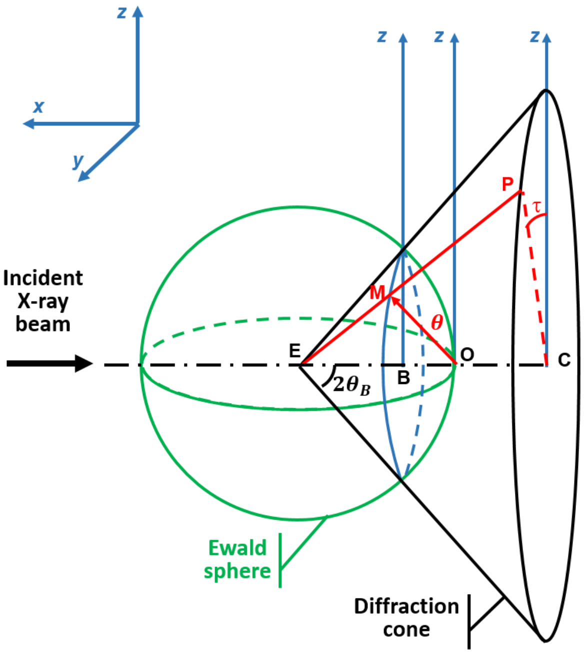


**Supplementary Fig. 1. Geometric description of the X-ray scattering experiment**. The laboratory frame is (*xyz*). An incident X-ray beam with wavelength $\lambda$ is antiparallel to the x-axis. The sample is at centre E of the Ewald sphere, and the radius is $2\pi/\lambda$. The origin of the reciprocal space is O, and wave-vector $\vec{Q}_{001}$, which gives the position of the Bragg peak *001* associated with a clay particle with orientation $\theta$ with respect to the *z*-axis, intersects the Ewald sphere at point M ($\vec{Q}_{001} = \overrightarrow{OM}$). By varying the platelet orientation, point M describes a circle of centre B on the Ewald sphere. The (EM) line intersects the detector plane (Cyz) at point P; $\overrightarrow{CP}$ makes an angle $\tau$ with the C*z* axis.

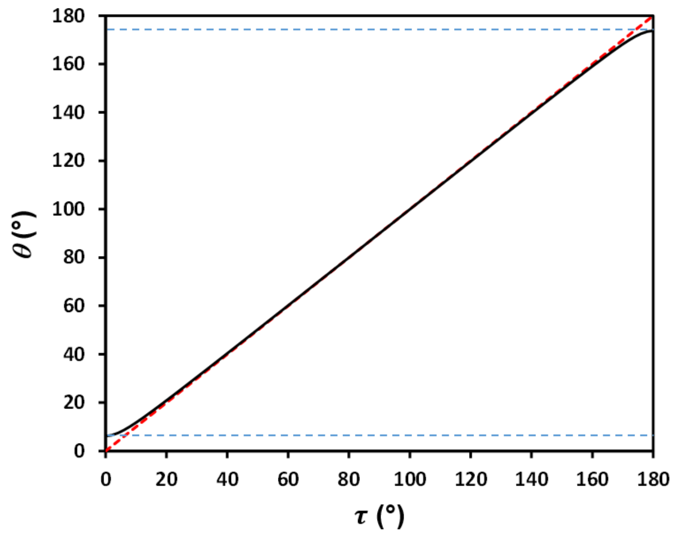


**Supplementary Fig. 2**. **Assessment of the $\theta$=$\tau$ approximation**. Black line: relationship between angles $\theta$ and $\tau$ from Supplementary Eq. (7). The dashed line in red corresponds to $\theta$=$\tau$. Horizontal blue dotted lines correspond to $\theta = \theta_B$ and to $\theta = 180° - \theta_B$.

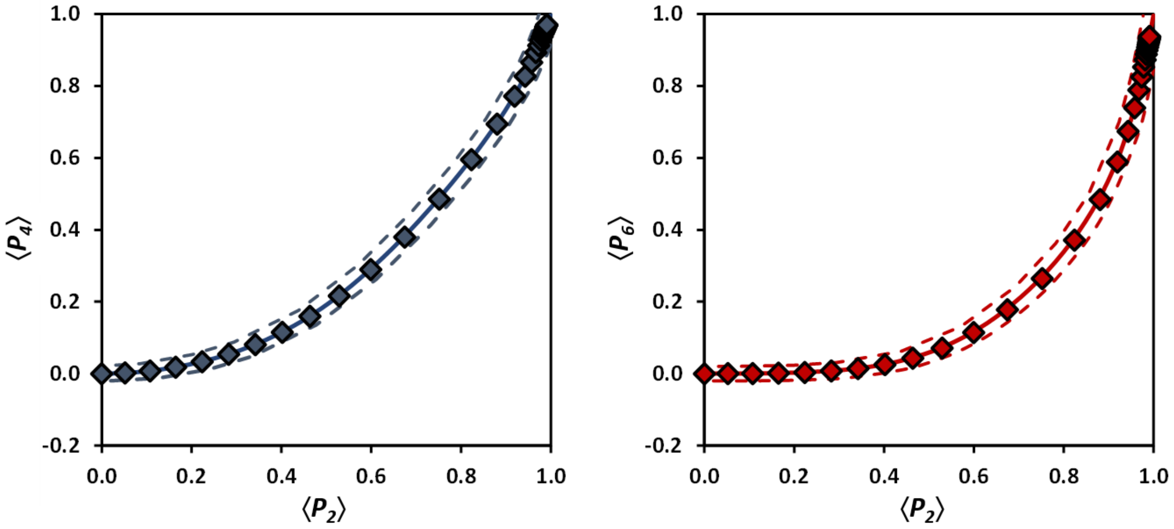


**Supplementary Fig. 3. Influence of the $\theta$=$\tau$ approximation on the calculated order parameter values**. The curve corresponding to the $\theta$=$\tau$ approximation is shown as a solid line. The dotted lines correspond to a confidence envelope of ±0.02 on $\langle P_2 \rangle$, $\langle P_4 \rangle$, and $\langle P_6 \rangle$ values for the $\theta$=$\tau$ case. Diamonds correspond to the case where no approximation is considered. Left: $\langle P_4 \rangle = f(\langle P_2 \rangle)$ and right: $\langle P_6 \rangle = f(\langle P_2 \rangle)$.

### Supplementary Note 2. Correlation between Lagrange multipliers $\lambda_2$ and $\lambda_4$.

Using Eqs. (19) and (20), we determined the values of the Lagrange multipliers $\lambda_2$ and $\lambda_4$ for pairs of experimental $<P_2>$ and $<P_4>$ values. The results are shown in Supplementary Fig. 4. The relation between $\lambda_2$ and $\lambda_4$ is well fitted with a power law of the form $\lambda_4$=A$(\lambda_2)^B$ with A=0.005±0.002 and B=5±0.4, and the general $f_{CM}(\theta)$ ODF for clay minerals (Eq. (21)) can be expressed as a function of a single parameter, $\lambda_2$.

As an example of the calculation of $f_{CM}(\theta)$ performed using Eq. (21), a $\lambda_2$ value of 1.00 leads to $\langle P_2\rangle$=0.22 and $\langle P_4\rangle$=0.03, whereas for $\lambda_2$=2.70, $\langle P_2\rangle$=0.65 and $\langle P_4\rangle$=0.35.

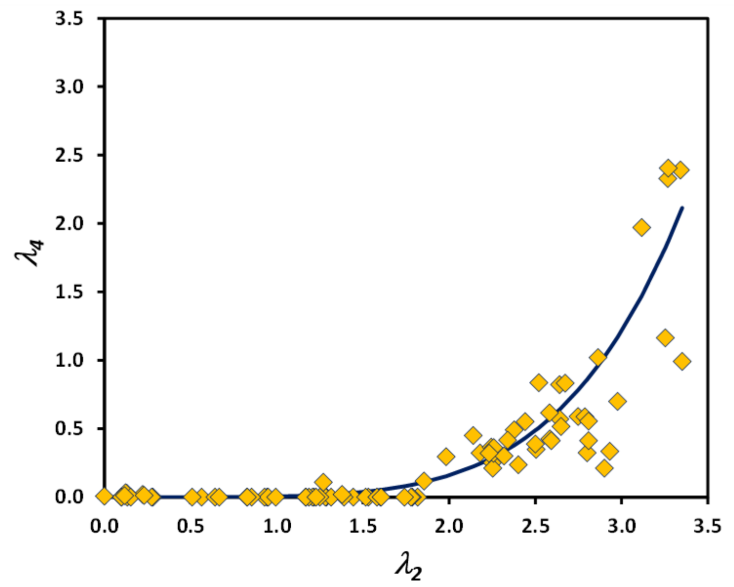


**Supplementary Fig. 4. Correlation between $\lambda_2$ and $\lambda_4$ Lagrange multipliers.** Experimental data points and the best fitted correlation of $\lambda_4$=0.005$(\lambda_2)^5$ used in Eq. (21) are shown as orange symbols and a solid curve, respectively.

### Supplementary Note 3. Experimental assessment of sample slicing along the main stratigraphic direction.

A random selection of 8 samples with various degrees of anisotropy was used to validate the assumption that the X-ray scattering (XRS) measurements were performed with the preferred orientation axis perpendicular to the incident X-ray beam, as drawn in Fig. 1b and Supplementary Fig. 1. To do so, two slices were prepared in the longitudinal and transverse direction to the poly(tetrafluoroethylene) cylinders, assuming that the preferential orientation axis is the axis of the cylinder. The sample slices were mounted on a goniometer head. All samples were kept perpendicular to the incident X-ray beam (Supplementary Fig. 5a). The first slice provides the description of preferential orientation, whereas the second slice should, in principle, show isotropic features according to the transverse isotropy of clay media. Supplementary Fig. 5b and 5c show the corresponding scattering pictures on the detector as well as the angular dependence of the XRS intensities at $Q = 0.88\text{Å}^{-1}$ (Fig. 1) on the *001* diffraction ring of a centrifuged kaolinite. The intensity of the *001* diffraction ring is strongly modulated for the slice prepared in the longitudinal direction of the cylinder; the sample presents a relatively high anisotropy degree ($\langle P_2\rangle$~0.6). The intensity along the *001* diffraction ring is constant for the slice prepared in the transverse direction. This result shows that the sample preparation and cutting process allow retrieval of

the main symmetry axis of the clay-rich media, thus fully justifying the methodology used to investigate the preferred orientation of clay platelets.

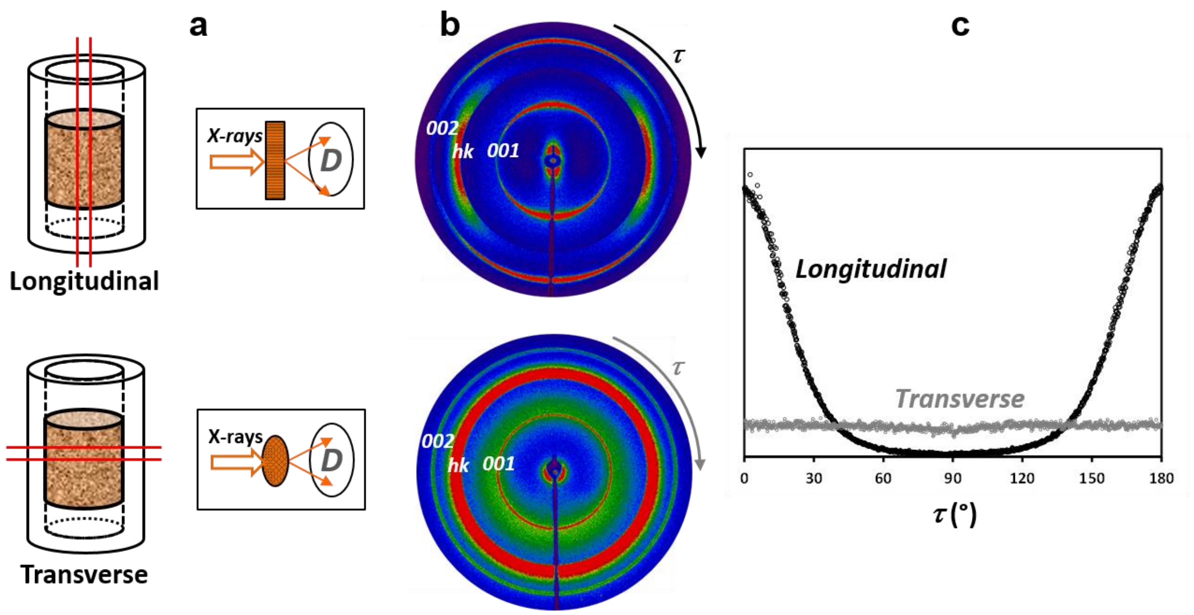


**Supplementary Fig. 5**. **Experimental assessment of sample slicing along the main stratigraphic direction**. **a** Sample slices in the longitudinal (top) or transverse (bottom) directions for the XRS analysis (D: detector). **b** Experimental XRS patterns obtained for the two sample slices (same notation as that in Fig. 1c). **c** The experimental ODFs deduced from the angular scan of the *001* diffraction ring using Eq. (4).
